# A high-throughput virus-induced gene silencing protocol identifies genes involved in multi-stress tolerance

**DOI:** 10.1186/1471-2229-13-193

**Published:** 2013-12-01

**Authors:** Venkategowda Ramegowda, Muthappa Senthil-kumar, Makarla Udayakumar, Kirankumar S Mysore

**Affiliations:** 1 Plant Biology Division, The Samuel Roberts Noble Foundation, 2510 Sam Noble Pkwy., Ardmore, OK 73402, USA; 2 Department of Crop Physiology, University of Agricultural Sciences, GKVK, Bangalore 560 065Karnataka, India; 3 Present address: VR: Department of Crop, Soil and Environmental Sciences, University of Arkansas, Fayetteville, AR 72701 USA; MS: National Institute of Plant Genome Research, Aruna Asaf Ali Marg, New Delhi 110 067, India

**Keywords:** Stress tolerance, Drought, Salinity, Temperature stress, Nonhost resistance, Bacterial pathogens, VIGS, PTGS, Translational genomics

## Abstract

**Background:**

Understanding the function of a particular gene under various stresses is important for engineering plants for broad-spectrum stress tolerance. Although virus-induced gene silencing (VIGS) has been used to characterize genes involved in abiotic stress tolerance, currently available gene silencing and stress imposition methodology at the whole plant level is not suitable for high-throughput functional analyses of genes. This demands a robust and reliable methodology for characterizing genes involved in abiotic and multi-stress tolerance.

**Results:**

Our methodology employs VIGS-based gene silencing in leaf disks combined with simple stress imposition and effect quantification methodologies for easy and faster characterization of genes involved in abiotic and multi-stress tolerance. By subjecting leaf disks from gene-silenced plants to various abiotic stresses and inoculating silenced plants with various pathogens, we show the involvement of several genes for multi-stress tolerance. In addition, we demonstrate that VIGS can be used to characterize genes involved in thermotolerance. Our results also showed the functional relevance of *NtEDS1* in abiotic stress, *NbRBX1* and *NbCTR1* in oxidative stress; *NtRAR1* and *NtNPR1* in salinity stress; *NbSOS1* and *NbHSP101* in biotic stress; and *NtEDS1*, *NbETR1*, *NbWRKY2* and *NbMYC2* in thermotolerance.

**Conclusions:**

In addition to widening the application of VIGS, we developed a robust, easy and high-throughput methodology for functional characterization of genes involved in multi-stress tolerance.

## Background

Understanding the physiological and molecular mechanism of abiotic stress tolerance in plants is the foundation for developing stress tolerant plants. Transcriptome analyses have been successfully used to identify genes associated with abiotic stress responses. A large number of genes altered during various abiotic stresses have been identified through expression profiling, expressed sequence tags (ESTs), and cDNA library generated from various plant species [1-6]. However, identifying the functional significance of individual differentially expressed genes during abiotic stresses is a daunting task. It is important to study the function of these stress-responsive genes not only to understand the mechanism of tolerance, but also for selecting candidate genes for improving the tolerance of susceptible species by genetic engineering.

Various functional genomics tools for gene overexpression or down-regulation have been developed for dissecting gene function [7]. Most commonly used gene knockout or knockdown approaches include ethyl methanesulfonate-induced point mutation [8], T-DNA insertion mutation [9], mutation by transposable element insertion [10], targeting induced local lesions in genomes [11] and gene silencing by RNAi [12]. Though these approaches have been widely and successfully used in functional analyses of genes, they have several disadvantages. For example, these approaches require generation of large-scale mutant populations or transgenic plants to screen for mutation in a gene of interest, which is a tedious and time consuming process. Mutation or insertion in a gene may not generate obvious phenotypes due to presence of gene families and gene duplications in plant genomes [13]. Virus-induced gene silencing (VIGS) is another functional genomics tool that avoids many of these limitations. In addition, VIGS can silence either an individual gene or multiple genes in a single plant and can also be used in a high-throughput manner to silence genes in multiple plants [14].

The basic principle of VIGS involves delivering viral RNA or DNA containing a partial sequence of a specific gene into plants [15]. Exogenous sequences are inserted into specific locations in the virus genome without destroying its infectivity [16]. The recombinant virus multiplies and spreads from the site of infection into new developing regions and triggers post-transcriptional gene silencing (PTGS; [17][16]). VIGS has been used as both a forward and reverse genetics tool to study gene function in plants [18]. Currently about 35 different VIGS vectors are available for gene silencing [18], of which the *Tobacco rattle virus* (TRV)-based VIGS vector is the most widely used silencing vector [19].

VIGS has been used as a tool for dissecting mechanisms of abiotic stress tolerance. Genes involved in tolerance to stresses, namely drought [20-24], UV [25], salinity [23,26], hypoxia [27] and oxidative stress [23], have been effectively silenced by VIGS, and their relevance under respective stresses was studied by various research groups. However, VIGS has not been used to identify genes that play various roles in abiotic stress tolerance through a high-throughput forward genetics screening. Similarly, despite its potential, VIGS has not been used to study the role of a particular gene under multiple stresses at the same time. This is mainly due to lack of a suitable protocol to coincide respective abiotic stresses with VIGS-mediated transient gene silencing as well as the absence of robust and easy methods to study the stress effect in silenced plants. Most of the methods currently used involve exposure of the whole plant to abiotic stress [24], which is a tedious process for large-scale screening. This scenario necessitates the requirement of high-throughput methodology to study stress effects on plants. In this manuscript, we describe a VIGS-based high-throughput methodology to study abiotic stress tolerance. Using this methodology, apart from confirming the role of several genes in biotic or abiotic stress tolerance which has been previously reported, we identified few genes that play a role in multi-stress tolerance.

## Results and discussion

### VIGS can continue to occur in excised leaf disks for more than six weeks

**Figure 1:**
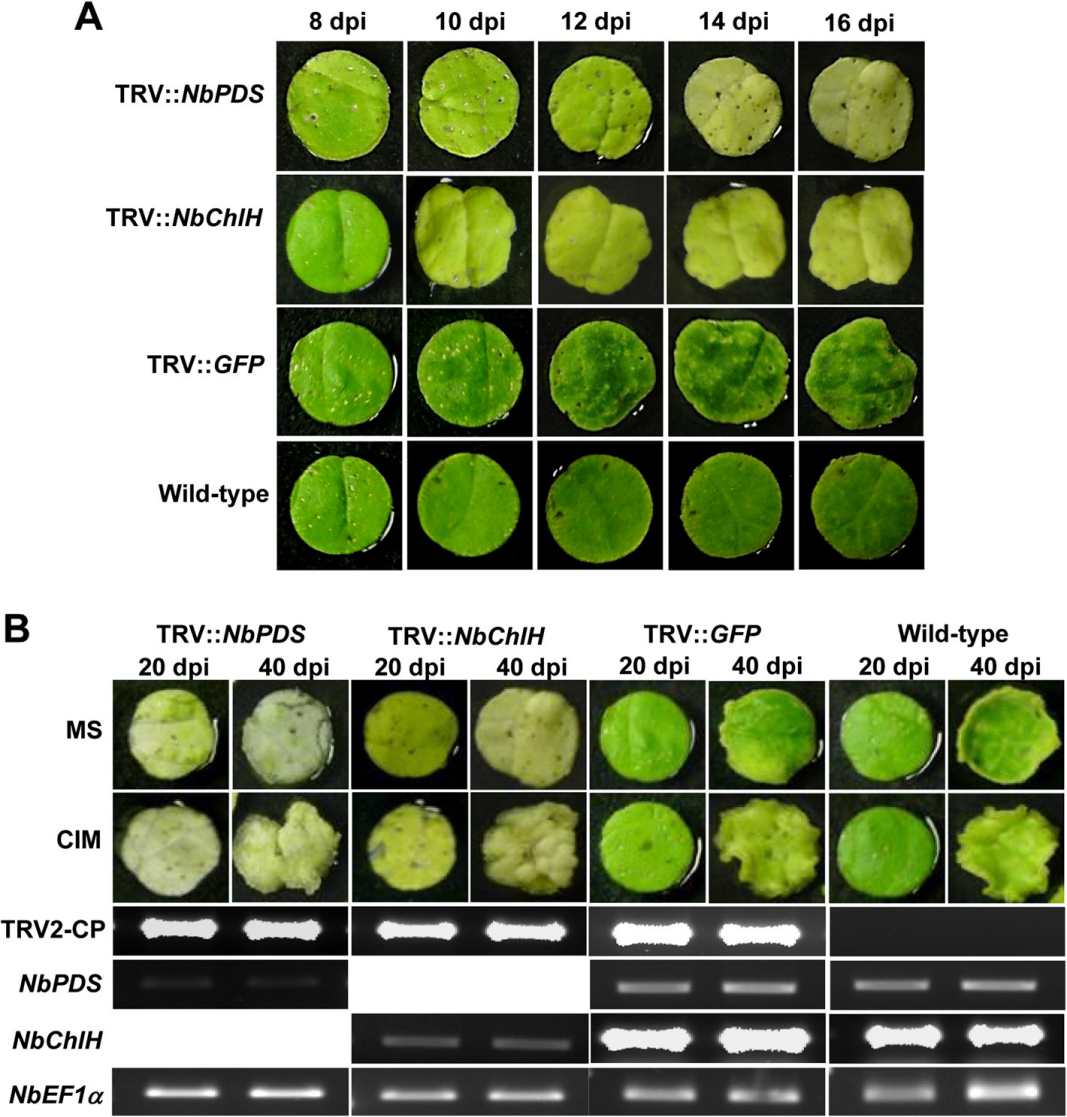
**Progression and persistence of VIGS in excised leaf disks*.*** Three-week-old *N. benthamiana* plants were inoculated with *Agrobacterium* carrying TRV::*NbPDS* and TRV::*NbChlH* constructs and grown in the greenhouse along with TRV::*GFP* (vector control) and wild-type non-inoculated plants. **A)** Leaf disks were collected from upper non-inoculated newly developed leaves at 8 dpi and incubated on MS medium. Since silencing of *NbPDS* and *NbChlH* genes in leaves is expected to produce discoloration, change in phenotype (from dark green to white or yellowing) was monitored and photographs were taken at two-day intervals. **B)** Leaf disks were collected from upper non-inoculated newly developed leaves from silenced plants at 20 dpi and incubated on MS medium and CIM. Persistence of silencing phenotype (white or yellow) was monitored for 20 more days, and photographs were taken at 20 and 40 dpi. The upper panel shows the respective phenotype and the lower panel shows RT-PCR that was performed using first-strand cDNA as a template, synthesized from total RNA (2 μg) primed by oligo-(dT)_15_, with 30 PCR cycles. Plates containing leaf disks were maintained under day/night period of 16 h/8 h at light intensity of 100 μmol m^-2^ s^-1^ in both cases. *NbPDS*, *N. benthamiana phytoene desaturase*; *NbChlH*, *N. benthamiana Mg-chelatase H subunit*; GFP, *green fluorescent protein*; dpi, days post-inoculation; MS, Murashige and Skoog medium; CIM, callus induction medium; TRV2-CP, *Tobacco rattle virus* RNA2 coat protein; *EF1α*, *Elongation factor-1 alpha* as loading control.

To determine whether the progression of VIGS can continue in excised leaf disks as it does in the whole plant, the leaf disks were collected from TRV::*NbPDS*-, TRV::*NbChlH*-, and vector control plants at 8 days post-inoculation (dpi) and incubated on MS medium. Similar treatment was also done for leaf disks from the non-inoculated wild-type plants. The progression of gene silencing was monitored until 16 dpi. *Agrobacterium*-mediated virus construct delivery into plant cell and initiation of VIGS is expected to take at least a week’s time. We, therefore, expected to see a silencing phenotype two weeks after TRV inoculation. Silencing of *NbPDS* and *NbChlH* genes in plants produces photobleaching and yellowing of leaves, respectively [28]. At 8 dpi, all the leaf disks were green, and the expected silencing phenotype started from 14 dpi and continued for few weeks (Figure 1A).

VIGS of both *NbPDS* and *NbChlH* genes occurred in leaf disks incubated both on MS medium and callus induction medium (CIM). In addition, uniform silencing persisted even up to 40 dpi. As expected, the silencing did not occur in vector control and non-inoculated wild-type plants (Figure 1B, upper panel). RT-PCR analysis of leaf disks maintained on CIM showed reduced transcript levels of silenced marker genes. All through the observed period, TRV was present in the leaf disks as indicated by PCR amplification of gene encoding coat protein (CP) (Figure 1B, lower panel). The presence of VIGS vector is essential for gene silencing by RNA viruses [29]. Conclusively, our results showed that gene silencing can occur in the excised leaf disks. These results are consistent with previous studies that used detached leaves from VIGS plants to study *Agrobacterium*-mediated plant transformation [30] and stress assays [24,26,31,32].

### Silencing genes involved in abiotic stress tolerance in excised leaf disks and their responses to various stresses

One of the objectives of this study was to develop a high-throughput protocol for identification and functional characterization of genes involved in imparting stress tolerance. We selected 28 *Nicotiana benthamiana* or tobacco homologous genes that were shown to be differentially expressed during various stress responses in different plant species (Table 1). GENEVESTIGATOR [33] data showed corresponding Arabidopsis homologs of these genes were also induced under respective stresses in Arabidopsis (data not shown). Fragments (300–580 bp) of these genes were individually cloned into *pTRV2* VIGS vector [19], and the viral constructs were used to silence individual corresponding genes in *N. benthamiana* as described previously [28,32,34]. Leaf disks were punched from the upper newly developed leaves at 20 dpi and the excised tissues were exposed to different abiotic stresses by incubating them on MS medium or CIM supplemented with respective stress-inducing agents. Responses of the gene-silenced leaf disks to individual abiotic stresses are given below.

**Table 1:** List of genes associated with various abiotic stresses and disease resistance used in the study

	**Gene**	**Functional annotation of gene fragments**	**NCBI GenBank accession #**	Reference*
**Salinity tolerance**				
1	*NbSOS1*	Salt overly sensitive 1 (Na+/H + antiporter)	JK739016	[35]
**Oxidative stress tolerance**				
2	*NbAPX3*	Ascorbate peroxidase 3	JK739005	[36]
3	*NbGST1*	Glutathione S-transferase 1	JK739012	[37,38]
4	*NbCAT3*	Catalase isozyme 3	JK739008	[39]
5	*NbDHAR1*	Dehydroascorbate reductase 1	JK739010	[40]
6	*NbGPX2*	Glutathione peroxidase 2	JK739011	[38,41]
7	*NbFER2*	Ferritin 2	JK739023	[42]
**High or low temperature stress tolerance**				
8	*NbHSP101*	101 kDa heat shock protein	JK739013	[43]
9	*NbBIP5*	Binding protein 5	JK739006	[44]
**Water deficit stress tolerance**				
10	*NbP5CS1*	Delta-1-pyrroline-5-carboxylate synthetase 1	JK739014	[45]
11	*NbCBL1*	Calcineurin B-like protein 1	JK739022	[46,47]
12	*NbMYC2*	MYC-related transcription factor 2	JK739021	[48]
13	*NbGBP16*	Putative monomeric G-protein 16	JK739018	[49]
14	*NbRBX1*	Ring box 1 like protein	JK739020	[50]
**Disease resistance**				
15	*NbPAL1*	Phenylalanine ammonia-lyase 1	JK739025	[51]
16	*NtEDS1*	Enhanced disease susceptibility 1	AF480489	[52]
17	*NtNPR1*	Non-expresser of pathogenesis related gene 1	AF480488	[53]
18	*NtRAR1*	Required for Mla12 resistance 1	AF480487	[54]
19	*NbADR1*	Activated disease resistance 1	JK739004	[55]
**Other genes up- or down-regulated by various stresses**				
20	*NbMC*	Metacaspase type II	JK739007	[56]
21	*NbCYCD2*	Cyclin D2.1 protein	JK739009	-
22	*NbCTR1*	Constitutive triple response 1-like protein kinase	JK739017	[57]
23	*NbWRKY1*	WRKY DNA-binding protein 1	AY547498	[57]
24	*NtMEK1*	MAP kinase/ERK kinase 1	AJ302651	[57]
25	*NbWRKY2*	WRKY DNA-binding protein 2	AY547495	[57]
26	*NbETR1*	Ethylene response 1	EF203416	[58]
27	*NbFLS1*	Flavonol synthase 1	JK739019	[59]
28	*NbMYB1*	MYB-related transcription factor 1	JK739024	[57]

*citations are given to indicate the gene function and not silencing of respective gene by VIGS.

#### Dehydration and osmotic stress

**Figure 2:**
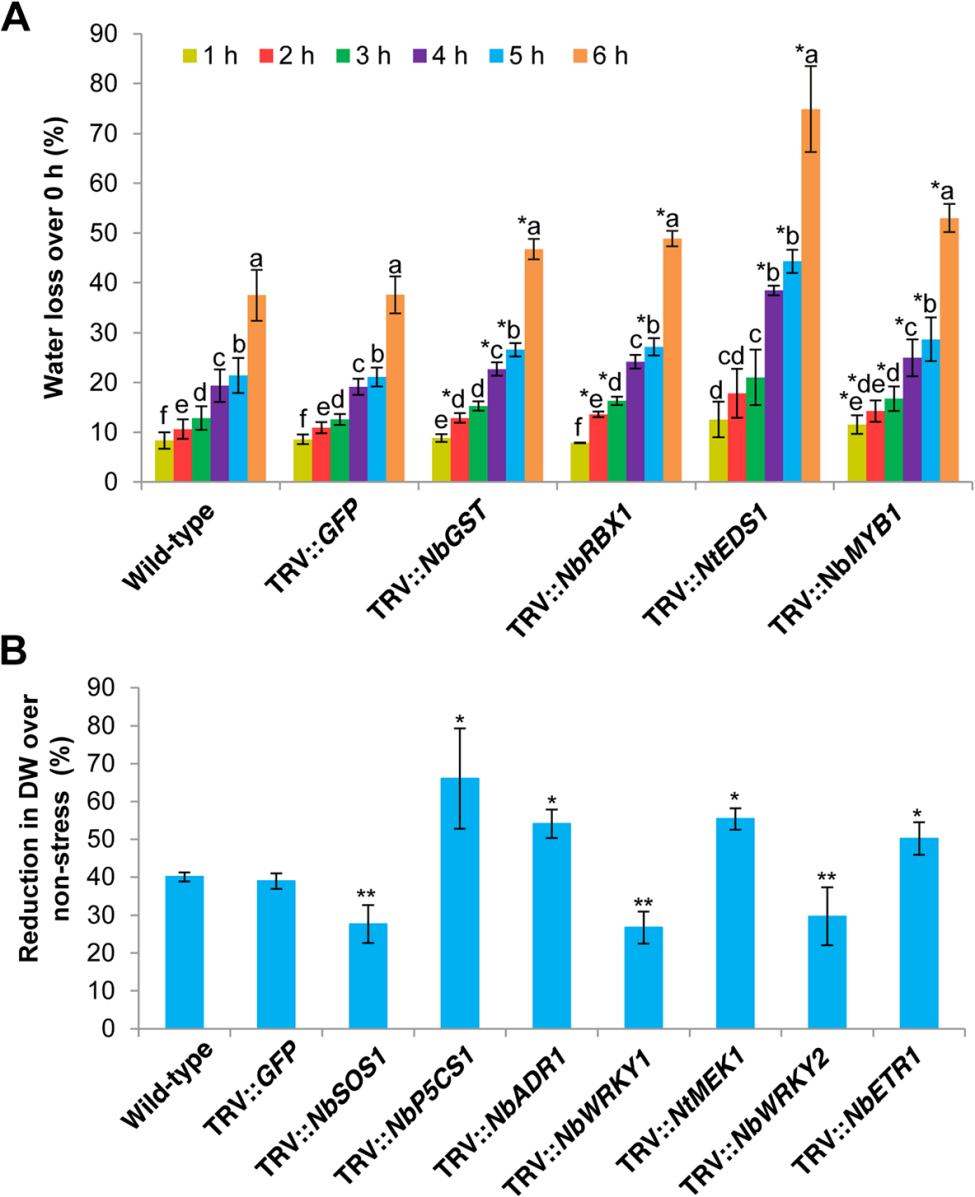
**Dehydration and osmotic stress tolerance of various gene-silenced leaves and leaf disks.** Three-week-old *N. benthamiana* plants were inoculated with TRV carrying inserts of a gene of interest and grown in the greenhouse along with TRV::*GFP* (vector control) and wild-type non-inoculated plants. **A)** Two fully expanded newly developed top leaves along with petiole were detached from these plants at 20 dpi and left on the bench. Fresh weight of these leaves was measured at the indicated time points. Decline in fresh weight was calculated and represented as percent water loss over their initial fresh weight. **B)** Leaf disks were collected from upper non-inoculated newly developed leaves from these plants at 20 dpi and incubated on CIM supplemented with 10% PEG for 20 days under the environmental conditions described in Figure 1. For non-stress, leaf disks were maintained on CIM without PEG. The effect of PEG-induced osmotic stress on callus growth was assessed by measuring callus dry weight at the end of the stress period. Each bar value represents mean ± sd (n = 12) of three independent experiments. ^a, b, c, d, e, f^ indicates that values are significantly different among time points within the plant type (ANOVA), and values significantly different from wild-type and vector control plants at that time point were indicated by ‘*’. Silenced plants which showed significant tolerance were indicated by ‘**’, and the rest were non-significant (Student’s *t*-test, *P* < 0.05).

Dehydration stress response was studied by estimating the rate of water loss during dehydration treatment. Detached leaf drying assay was reported to be an easy assay for testing large numbers of plants for their dehydration stress avoidance [60]. By utilizing this assay, we studied the responses of gene-silenced plants to dehydration stress by evaluating the decline in fresh weight of detached leaves. Among 28 different gene-silenced plants, leaves from *NbGST*-, *NbRBX1*- and *NbMYB1*-silenced plants showed a 10-20% increase in water loss over vector control plants at 6 h (Figure 2A). Our results agree with previous reports that *GST*, *RBX1* and *MYB1* genes are involved in water deficit stress tolerance [50,61,62] and indicate that our method can be used to identify genes involved in dehydration stress avoidance. Interestingly, leaves harvested from *NtEDS1*-silenced plants showed about 40% increase in water loss over the control at 6 h after detachment from plants (Figure 2A). This data indicate that the *EDS1* gene, whose function was implicated mainly in disease resistance [63], may also function in dehydration avoidance.

Osmotic stress response was studied by imposing constant low water potential under non-transpiring conditions using polyethylene glycol (PEG). Callus growth on the PEG-stressed leaf disks was measured. Leaf disks from *NbP5CS1*-, *NbADR1*-, *NtMEK1*- and *NbETR1*-silenced plants showed about 10-20% more reduction in callus growth compared to control plants (Figure 2B). This finding supports previous reports that *P5CS1*, *ADR1*, *MEK1* and *ETR1* genes play a role in osmotic stress tolerance [45,64-66]. Interestingly, leaf disks from the *NbSOS1*-, *NbWRKY1*- and *NbWRKY2*-silenced plants showed significant tolerance to PEG stress (Figure 2B). Although *SOS1*, *WRKY1* and *WRKY2* genes were previously implicated in imparting abiotic or biotic stress tolerance [35,57,67], it is possible that these genes are not playing positive roles under osmotic stress. VIGS has been previously shown to be a potential method for studying the relevance of only a few genes under water deficit stress [21,32] because the stress imposition methods used were cumbersome and not feasible for large-scale experiments. However, the results described here show that this lacuna can now be overcome. Taken together, our data suggest that leaf/leaf disks undergoing VIGS can be used to identify and characterize gene functions in dehydration and osmotic stress tolerance in a high-throughput manner.

#### Salinity stress

**Figure 3:**
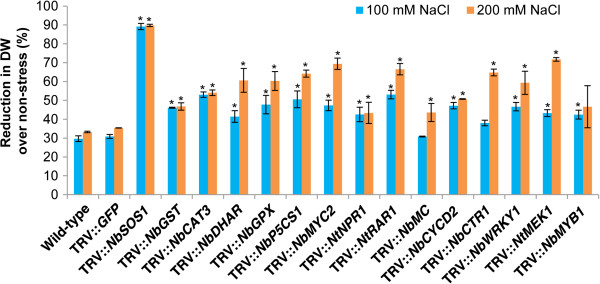
**Salinity tolerance of gene-silenced leaf disks.** Three-week-old *N. benthamiana* plants were inoculated with TRV carrying inserts of a gene of interest and grown in the greenhouse along with TRV::*GFP* (vector control) and wild-type non-inoculated plants. Leaf disks were collected from these plants at 20 dpi. Leaf disks were incubated on CIM supplemented with either 100 or 200 mM NaCl for 20 days under the environmental conditions described in Figure 1. For non-stress, leaf disks were maintained on CIM without NaCl. Callus dry weight was measured at the end of the stress period. Each bar value represents mean ± sd (n = 12) of three independent experiments. Values significantly different from wild-type and vector control plants were indicated by ‘*’, and the rest were non-significant (Student’s *t*-test, *P* < 0.05).

In order to develop a high-throughput protocol for assessing gene function in salinity tolerance, we incubated gene-silenced leaf disks on CIM with NaCl followed by callus growth assessment. Leaf disks from *NbSOS1*-silenced plants showed the highest reduction (~90%) in growth, both under 100 and 200 mM NaCl stress, compared to non-stressed plants (Figure 3) while wild-type and vector control plants showed only ~30% reduction compared to non-stressed plants. Since the Arabidopsis *SOS1* gene has been very well shown to impart salinity tolerance [35], this data validates the usefulness of this protocol for salinity tolerance studies. Leaf disks from *NbP5CS1*-*, NbMYC2*-*, NtRAR1*-, *NbCTR1*- and *NtMEK1*-silenced plants showed about a 60% reduction in growth over corresponding non-stressed plants. Further, leaf disks from *NbGST*-, *NbCAT3*-, *NbDHAR-*, *NbGPX-*, *NtNPR1*-, *NbMC*-, *NbCYCD2-*, *NbWRKY1*- and *NbMYB1*-silenced plants also showed around 40-50% reduction in growth, suggesting the relevance of these genes in salinity tolerance. Involvement of *P5CS1*, *MYC2, CTR1*, *MEK1*, *CAT*, *DHAR, GPX*, *NPR1*, *CYCD2, WRKY1* and *MYB1* genes in salinity tolerance has been shown earlier [48,64,68-72]. In addition to these, our results also suggest the plausible role of *MC* in salt tolerance. Another interesting observation that we made was the higher susceptibility of *NtRAR1*-silenced plants to salinity, a gene mainly implicated in disease resistance [54], indicating that *RAR1* may play a positive role under salinity stress.

#### High and low temperature stress

**Figure 4:**
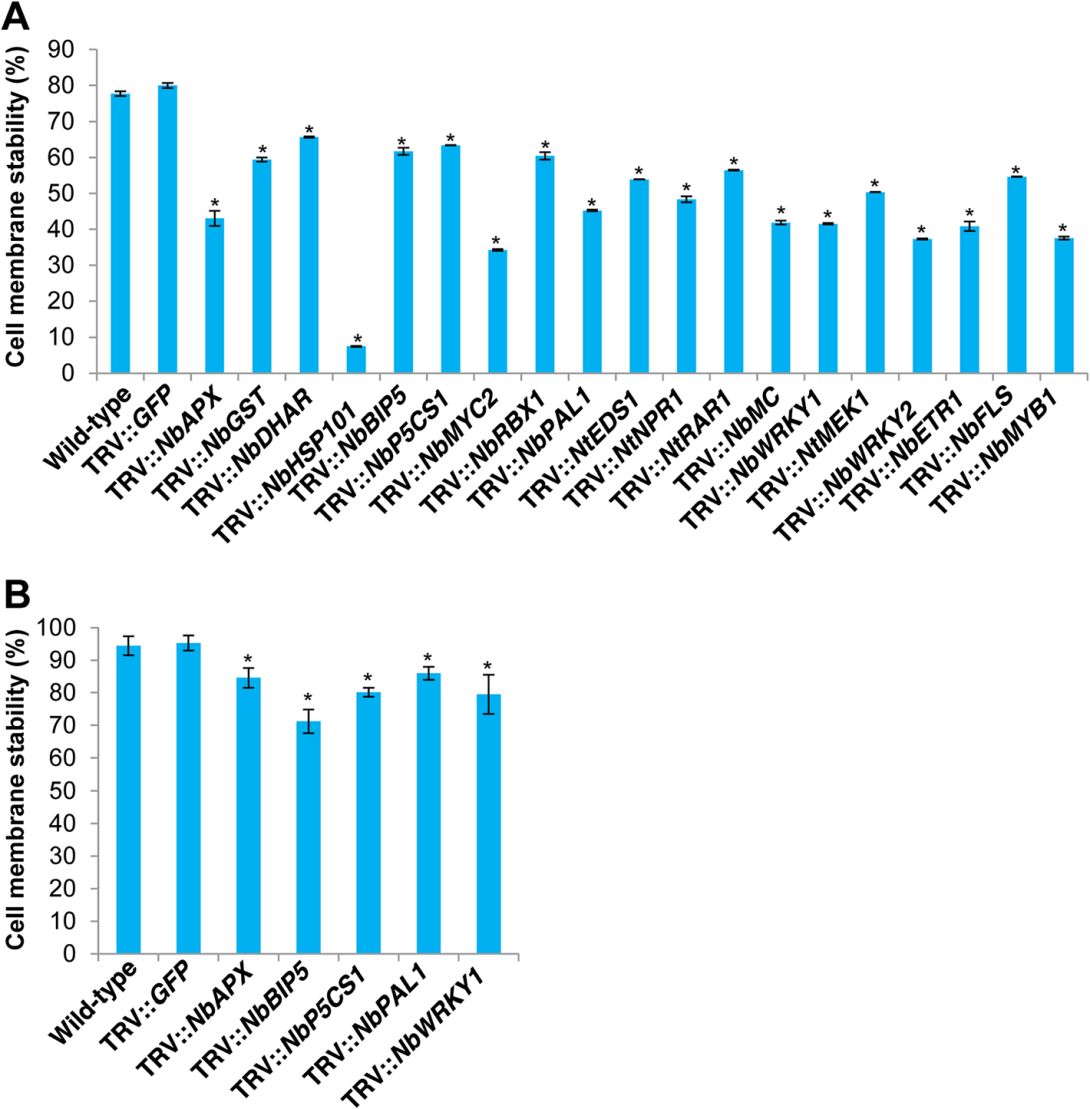
**High and low temperatures stress tolerance of leaf disks from gene-silenced plants.** Three-week-old *N. benthamiana* plants were inoculated with TRV carrying inserts of a gene of interest and grown in the greenhouse along with TRV::*GFP* (vector control) and wild-type non-inoculated plants. Leaf disks were collected from these plants at 20 dpi and floated on deionized water and exposed to either high or low temperature stress. **A)** For high temperature stress, leaf disks were initially incubated at 35°C for 6 h and then at 45°C for 1 h. Another set of disks was maintained under non-stress conditions. Electrical conductivity of the medium was measured at the end of the stress period. **B)** For low temperature stress, leaf disks were initially incubated at 4°C for 12 h and then at -2°C for 1 h. Another set of samples was maintained under non-stress conditions. Electrical conductivity of the medium was measured at the end of the stress period. Based on electrical conductivity values, cell membrane stability was calculated. Each bar value represents mean ± sd (n = 12) of three independent experiments. Values significantly different from wild-type and vector control plants were indicated by ‘*’, and the rest were non-significant (Student’s *t*-test, *P* < 0.05).

High and low temperature stress tolerances of gene-silenced and control plants were assessed by measuring cell membrane stability (CMS) [73]. Under high temperature stress (45°C), leaf disks obtained from *NbHSP101*-silenced plants showed a high reduction (90%) in CMS while the leaf disks from vector control plants had only 20% reduction (Figure 4A). This reduction in CMS in silenced plants was highly correlated with reduced transcript levels of *HSP101*. Semi-quantitative RT-PCR results showed 3.8-fold reduction in *HSP101* transcripts in silenced plants when compared to vector control plants (Additional file 1). Several gene-silenced leaf disks also showed significant reduction in their membrane stability. Among them, about 60% reduction was found in the leaf disks from the *NbAPX*-, *NbMYC2*-, *NbMC*-*, NbWRKY1*-*, NbWRKY2*- and *NbETR1*-silenced plants. This data indicate that *NbHSP101* plays an important role in thermotolerance and is consistent with the previous reports [74]. Furthermore, our results show that the genes like *MYC2*, *WRKY2* and *ETR1* that are known to play a role in various other abiotic stresses [48,66,67] might also play a role in thermotolerance.

Under low temperature stress (-2°C), leaf disks from *NbAPX*-*, NbBIP5*-, *NbP5CS1*-*, NbPAL1*- and *NbWRKY1*-silenced plants showed higher membrane damage compared to control plants (Figure 4B). However, CMS of leaf disks from other gene-silenced plants studied did not significantly differ from the vector control plants. *NbAPX*-, *NbBIP5*-, *NbP5CS1*-, *NbPAL1*- and *NbWRKY1*-silenced plants showed susceptibility to both high and low temperature stresses, suggesting that these genes may have a general role in protecting the cell membrane during stresses.

#### Oxidative stress

**Figure 5:**
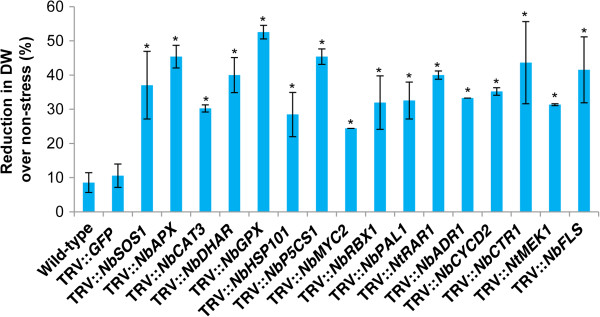
**Oxidative stress tolerance of leaf disks collected from gene-silenced plants.** Three-week-old *N. benthamiana* plants were inoculated with TRV carrying inserts of a gene of interest and grown in the greenhouse along with TRV::*GFP* (vector control) and wild-type non-inoculated plants. Leaf disks were collected from these plants at 20 dpi. Leaf disks were incubated on CIM supplemented with or without 10 μM menadione for 20 days under the environmental conditions described in Figure 1. Callus dry weight was measured at the end of the stress period, and the percent reduction over corresponding non-stressed samples was calculated. Each bar value represents mean ± sd (n = 12) of three independent experiments. Values significantly different from wild-type and vector control plants were indicated by ‘*’ and the rest were non-significant (Student’s *t*-test, *P* < 0.05).

Leaf disks from the gene-silenced plants were subjected to oxidative stress by incubating them on menadione-supplemented CIM for callus growth assay. Callus growth of leaf disks from *NbGPX*-silenced plants showed the largest reduction in weight (~52%) compared to vector control (~10%) (Figure 5). *GPX* has been previously implicated in regulating ROS during oxidative stress [37]. In addition, leaf disks from plants silenced for other known oxidative stress associated genes like *APX*, *CAT3* and *DHAR* also showed 30-45% growth reduction. Our results support previous findings that showed involvement of *APX*[75], *CAT*[76], *DHAR*[77], *SOS1*[78], *HSP101*[79], *P5CS1*[80], *MYC2*[81], *PAL1*[82], *MEK1*[65] and *FLS1*[83] in oxidative stress, thus validating the usefulness of this assay in studying genes associated with oxidative stress. Furthermore, among other assayed plants which showed significant reduction in growth, for the first time we suggest the involvement of *RBX1* and *CTR1* in oxidative stress. Taken together, we demonstrated that VIGS in combination with callus growth assay is useful for characterizing genes imparting oxidative stress tolerance.

### Assessing the response of gene-silenced plants to host and nonhost bacterial pathogen infection

Abiotic stress tolerance pathways adapted by plants are known to share a significant overlap with pathways involved in plant-pathogen interactions [84]. Hence, apart from abiotic stress tolerance, we also studied responses of the 28 gene-silenced plants to pathogen infection. Two leaves of each gene-silenced plant were spot-inoculated with either a host pathogen, *Pseudomonas syringae* pv. *tabaci*, or nonhost pathogens *P. syringae* pv. *tomato* T1 and *Xanthomonas campestris* pv. *vesicatoria* expressing *GFPuv*[85,86]. Growth of these bacterial pathogens in the inoculated spots was visualized using UV light under dark. Host pathogen-inoculated spots showed green fluorescent signals reflecting the bacterial colonies. Greater variation in host pathogen growth was observed among different gene-silenced plants (Table 2). Nonhost-pathogen-inoculated spots in the wild-type and the vector control plant leaves did not produce any green fluorescence signal as the nonhost resistance mechanism was fully functional in these plants. In addition to bacterial growth under UV light, visual observations were also made to note disease symptoms (Table 2). Apart from confirming the involvement of several genes such as *EDS1, RAR1* and *ADR1* in plant disease resistance as reported before, our results also suggest the plausible role of *SOS1* and *HSP101*, which are mainly associated with abiotic stresses, in biotic stress tolerance.

**Table 2:** Responses of gene-silenced leaf disks to abiotic stresses, and bacterial pathogen growth on silenced plants

**Name of silenced gene**	**Abiotic stresses**^**1**^					**Disease resistance**^**2 **^**(bacterial pathogens)**		
						**Host**	**Nonhost**	
	**Salinity**	**High temperature**	**Low temperature**	**Oxidative**	**Osmoticum**	**Pstab**	**PstT1**	**XCV**
WT	*	*	*	*	*	##	No	No
*TRV::GFP*	*	*	*	*	*	##	No	No
*NbSOS1*	++++	*	*	+	*	##	No	†
*NbAPX*	*	++	+	+++	*	##	†	No
*NbGST*	++	+	*	++	++	###	†	†
*NbCAT3*	++	*	*	++	*	###	†	†
*NbDHAR*	+	+	*	++	*	##	No	†
*NbGPX*	++	*	*	++++	*	##	†	No
*NbFER2*	*	*	*	*	*	##	†	No
*NbHSP101*	+	++++	*	++	*	##	No	†
*NbBIP5*	*	+	++	*	*	##	No	No
*NbP5CS1*	++	+	+	+++	++++	###	†	No
*NbCBL1*	*	+	*	+	*	##	No	No
*NbMYC2*	++	++	*	+	*	###	†	No
*NbGBP16*	*	*	*	*	*	##	No	No
*NbRBX1*	*	+	*	+	++	##	No	No
*NbPAL1*	*	++	+	++	*	##	†	No
*NtEDS1*	*	++	*	*	++++	###	†	†
*NtNPR1*	++	++	*	+	*	###	†	No
*NtRAR1*	++	+	*	+	*	###	†	†
*NbADR1*	*	+	*	+	+++	###	†	†
*NbMC*	+	++	*	*	*	##	No	No
*NbCYCD2*	++	*	*	++	*	###	†	†
*NbCTR1*	+	*	*	++	*	##	†	No
*NbWRKY1*	++	++	+	*	+	###	†	No
*NtMEK1*	++	+	*	++	+++	##	No	No
*NbWRKY2*	*	++	*	*	+	##	No	No
*NbETR1*	*	++	*	+	++	##	No	No
*NbFLS*	*	+	*	++	*	##	No	†
*NbMYB1*	++	++	*	+	++	##	No	No

^1^Abiotic stress phenotype was represented as ^*^leaf disks with no observable phenotypic changes when compared to wild-type; ^+^trivial discoloration; ^++^moderate discoloration with wrinkling; ^+++^complete discoloration and/or wrinkle with necrotic spots and ^++++^complete cell death.

^2^The disease symptom development on pathogen-inoculated leaves was scored as percent leaf area covered and represented as ^##^50% of the leaf area showing disease symptoms when inoculated with host pathogen Pstab (*Pseudomonas syringae* pv. *tabaci*); ^###^75% of the leaf area showing disease symptoms when inoculated with Pstab; ^No^leaves with no visible disease symptoms when inoculated with nonhost pathogens PstT1 (*P. syringae* pv. *tomato* T1) and XCV (*Xanthomonas campestris* pv. *vesicatoria*); ^†^up to 50% of leaf showing disease symptoms when inoculated with PstT1 and XCV.

### Identification of genes involved in multi-stress tolerance

Simulated drought, salinity and oxidative stress responses were tested by incubating leaf disks collected from gene-silenced, wild-type and vector control plants on MS medium supplemented with either PEG or NaCl or menadione, respectively. This assay was performed on MS medium for the convenience of identifying a clear phenotype, which will be difficult on CIM as callus induction and/or growth might interfere with the phenotype. Pathogen response was assessed by inoculating *GFPuv*-expressing host pathogen *P. syringae* pv. *tabaci* and two nonhost pathogens, *P. syringae* pv. *tomato* T1 and *X. campestris* pv. *vesicatoria*, and visualizing the bacterial growth by GFP fluorescence under UV light. Leaf disks from wild-type and vector control plants incubated on abiotic stress medium were healthy and green for about two weeks. However, leaf disks from gene-silenced plants showed various phenotypes including discoloration, spotted cell death and wrinkling. A representative phenotype of leaf disks from *NbSOS1*-silenced plants is shown in Additional file 2A. Similarly, during pathogen infection, wild-type non-inoculated and vector control plants did show disease symptoms to host pathogen *P. syringae* pv. *tabaci*, but severe disease symptoms were observed in some of the gene-silenced plants. Some of the gene-silenced plants also supported the growth of nonhost pathogens *P. syringae* pv. *tomato* T1 and *X. campestris* pv. *vesicatoria*, while no bacterial growth was observed in wild-type non-inoculated and vector control plants. A representative disease phenotype of *NbGPX*-silenced plants is shown in Additional file 2B. Phenotypic responses were recorded for each gene-silenced leaf disk/plant separately exposed to various different abiotic and biotic stresses (Table 2).

Most of the gene-silenced leaf disks showed susceptibility to salinity and oxidative stress (Table 2), indicating that these two stresses are regulated by a wide range of genes. Interestingly, only a few gene-silenced leaf disks were susceptible to low temperature stress. This may indicate the unique nature of this stress effect and reciprocating plant response. *NbGST*-, *NbWRKY1*-, *NtMEK1*- and *NbMYB1*-silenced leaf disks showed susceptibility to a wide range of stresses studied. *NbP5CS1*-silenced leaf disks were susceptible to all abiotic stresses studied. More interestingly, 11 out of 14 genes that were previously implicated to play a role in abiotic stress tolerance– when individually silenced the plants were more susceptible to at least one host or nonhost pathogen infection (Table 2). In addition to identifying new genes not previously implicated in biotic or abiotic stress tolerance, our results also confirmed the previous findings that showed the involvement of some of these genes in both biotic and abiotic stresses. For example, Arabidopsis activation tagged lines constitutively expressing *ADR1* exhibited resistance against broad spectrum virulent pathogens through SA-dependent activation of defense genes [55] and also showed tolerance to drought stress through SA-dependent activation of drought stress-related genes such as *DREB2A*[66]. The *ADR1* mediated drought tolerance in Arabidopsis needed *EDS1*[66], a gene that plays a role in disease resistance [63]. These results suggested the role of *ADR1* and *EDS1* in both biotic and abiotic stresses. Also in our study, both *ADR1*- and *EDS1*-silenced plants showed susceptibility to osmotic stress as well as pathogen infection. *GST* is also known to be involved in both biotic and abiotic stresses. Silencing of *NbGSTU1* in *N. benthamiana* showed significantly higher lesions and more colonization by *Colletotrichum orbiculare*[87]. The role *GSTs* during abiotic stress tolerance mainly through detoxification of ROS has been suggested. Overexpression of *GST* along with *GPX* in tobacco enhanced seedling growth under salt stress and also reduced oxidative damage [37]. In our study *GST1*-silenced plants showed susceptibility to salinity, oxidative stress, osmotic stress and pathogen infection thus confirming the role of *GST* in both biotic and abiotic stresses. These findings further suggest the existence of common mechanisms underlying tolerance to abiotic and biotic stresses. Other genes which are commonly required for tolerance to both abiotic and biotic stresses include regulatory genes like transcription factors. For example, the *NbWRKY1-*silenced leaf disks showed moderate susceptibility to salt, high temperature and PEG-induced osmotic stresses while *NbMYC2*-silenced leaf disks were moderately susceptible to salt, high temperature and oxidative stresses. In addition, these silenced plants were also more susceptible to host and nonhost pathogens. The other regulatory gene-silenced leaf disks like *NtRAR1*, *NtNPR1* and *NbCTR1* also showed susceptibility to both abiotic and biotic stresses. These results indicate that the high-throughput VIGS methodology used here to study abiotic stress tolerance can also be used to dissect mechanisms involved in multi-stress tolerance of plants.

### Validating the function of specific genes at the whole plant level

In order to validate the results obtained from the leaf disk assays, we chose four genes to confirm their role in stress tolerance at the whole plant level. *NbP5CS1*, *NtEDS1*, *NbHSP101* and *NbSOS1* genes were individually silenced in *N. benthamiana*. Down-regulation of target genes in respective gene-silenced plants was confirmed by semi-quantitative RT-PCR (Additional file 1). These gene-silenced plants were then individually subjected to various abiotic stresses at the whole plant level, and their responses were recorded as below.

#### Water deficit stress

*P5CS1* has been shown to impart water deficit stress tolerance in plants by facilitating enhanced biosynthesis and accumulation of proline [45]. Therefore, we selected *NbP5CS1*-silenced plants for imposing water deficit stress at the whole plant level. Similarly, the *AtEDS1* has been implicated in resistance against powdery mildew disease caused by *Erysiphe necator*[88] and water deficit stress tolerance [64]. Our results also showed that the *NtEDS1* gene is important for basal resistance against host pathogen *P. syringae* pv. *tabaci* (Table 2). Further, the leaf disk assay and detached leaf assay showed that *NtEDS1-*silenced plants were highly susceptible to osmotic stress (Table 2) and dehydration stress (Figure 2), respectively. Therefore, we also studied the response of *NtEDS1*-silenced plants under water deficit stress at the whole plant level.

**Figure 6:**
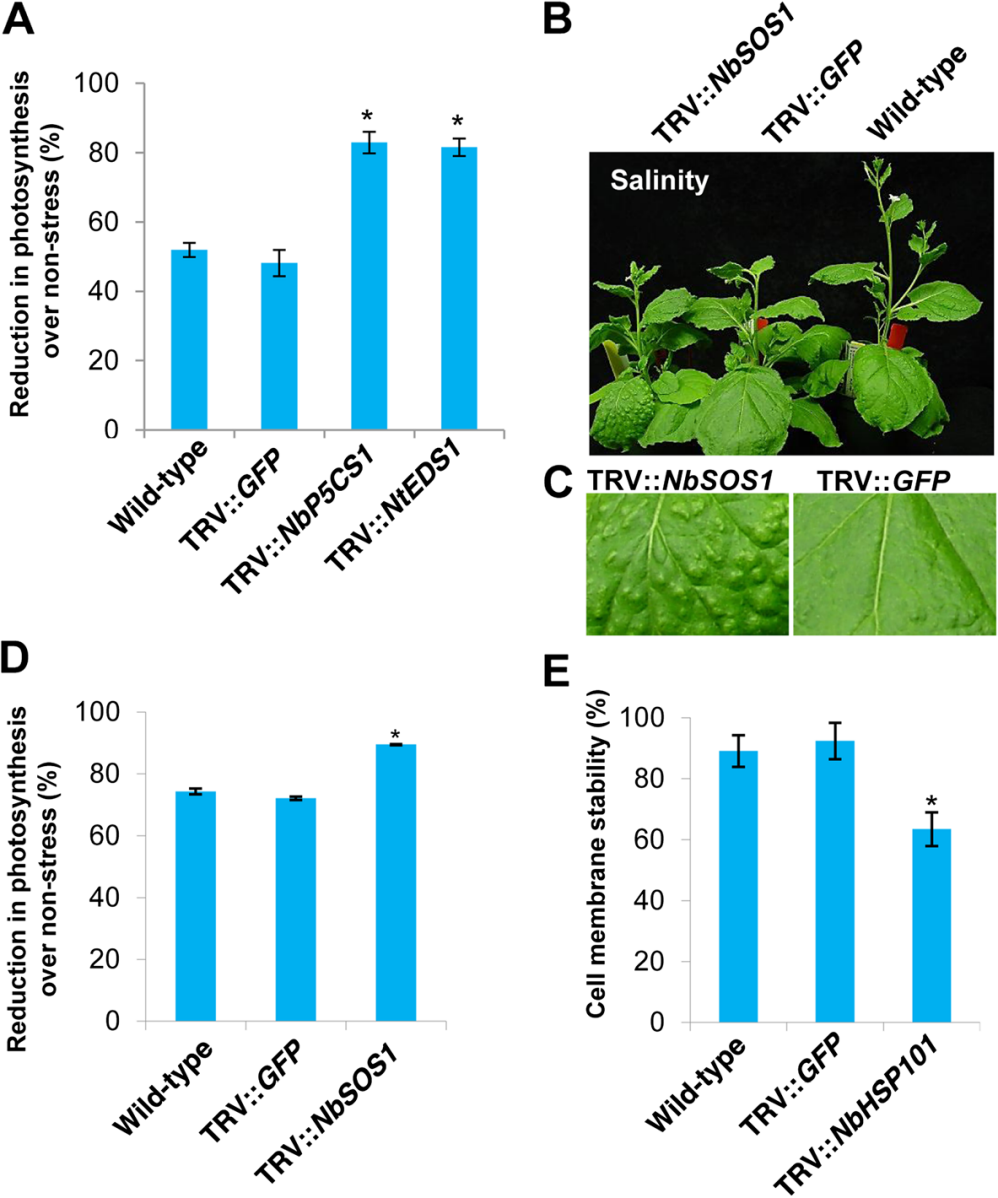
**Abiotic stress response of selected genes at the whole plant level.** Three-week-old *N. benthamiana* plants were inoculated with respective TRV derivatives, TRV::*GFP* and maintained in the greenhouse along with wild-type non-inoculated plants. **A)** Photosynthetic rates of *NbP5CS1*- and *NtEDS1*-silenced plants under water deficit stress. Stress was imposed on plants at 20 days after silencing by gradually bringing down the water level to 50% FC over a period of 15 days. Another batch of plants was maintained under non-stress conditions. Photosynthetic rates were measured in the third newly formed and fully expanded leaf. **B)** Effect of salt stress on *NbSOS1*-silenced plants. *NbSOS1-*silenced plants were irrigated with NaCl solution at 20 dpi while gradually exposing plants to higher NaCl concentration from 100 mM for 5 days, then 200 mM for 5 days and subsequently to 300 mM for 5 days. Another batch of plants was maintained under non-stress conditions. Photographs were taken at the end of the stress period. **C)** Leaf from *NbSOS1*-silenced plant showing salt pustules in the adaxial surface due to higher salt accumulation. **D)** Photosynthetic rates were measured at the end of the stress period. **E)** Effect of heat stress on cell membrane stability of *NbHSP101*-silenced plants. Wild-type, vector control and *NbHSP101*-silenced whole plants were initially acclimated with mild temperatures (35°C for 6 h) and subsequently exposed to high temperature stress (45°C for 1 h). Another batch of plants was maintained under non-stress conditions. Leaf disks were collected from these plants at the end of stress, and ion leakage was measured as described in the Materials and Methods section. Each bar value represents mean ± sd (n = 5) of three independent experiments. ‘*’ suggests values were significantly different from wild-type and vector control plants (Student’s *t*-test, *P* < 0.05).

Photosynthetic performance of *NbP5CS1*- and *NtEDS1*-silenced plants maintained under water deficit stress of 50% FC was measured. *NbP5CS1*- and *NtEDS1*-silenced plants independently showed more than 80% reduction in photosynthetic efficiency over non-stressed plants compared to only a 50% reduction in control plants (Figure 6A). These data suggest that the photosynthetic machinery is greatly impaired in these gene-silenced plants upon drought stress. Less reduction in photosynthetic efficiency under stress is one of the mechanisms adapted by stress tolerant plants to cope with the water deficit stress [89]. Hence, greater reduction in the photosynthetic efficiency in the *NbP5CS1*- and *NtEDS1*-silenced plants reflects the relevance of these two genes in water deficit stress tolerance. Taken together, these results validate the VIGS methodology developed for studying water deficit or dehydration or osmotic stress tolerance.

#### Salinity stress

We selected the *NbSOS1* gene in order to validate our salinity screening assays. *NbSOS1*-silenced whole plants were irrigated with saltwater by gradually increasing salt concentration from 100 to 300 mM at 5-day intervals. Salinity stress response was assessed at the end of the stress period by measuring photosynthesis. *NbSOS1* silenced plants under salt stress showed reduced growth compared to corresponding control plants, and no growth differences were observed under non-stress conditions (Figure 6B). Consistent with our observations, the growth of the *sos1* Arabidopsis mutant was inhibited by high Na + [35]. In addition to growth reduction, raised pustules (salt accumulated pockets) were observed on the adaxial surface of *NbSOS1*-silenced *N. benthamiana* leaves (Figure 6C). Since salt tolerance of plants depends on the ability to exclude Na + from the shoot and maintain a low cellular Na+/K + ratio [90], one of the reasons for the susceptibility of silenced plants to salt might be the disruption of Na + homeostasis in the cell. Salt has been previously shown to inhibit photosynthesis [91], and *SlSOS1* overexpression in tomato prevented Na + from reaching the photosynthetic tissues by extruding Na + out of the root and also retaining Na + in the stems [92]. Consistent with this, *NbSOS1*-silenced *N. benthamiana* plants exposed to salt stress in this study showed about 10% reduction in photosynthetic efficiency (Figure 6D).

#### High temperature stress

*NbHSP101*-silenced whole plants exposed to high temperature stress showed less membrane stability compared to corresponding vector control plants (Figure 6E). Consistently, Arabidopsis *hot* mutants with reduced thermotolerance, expressing lower levels of *AtHSP101*, had higher membrane damage under high temperature stress [93]. HSP101 is an important chaperon protein known to be involved in imparting thermotolerance in plants [94,95]. Overexpression of *AtHSP101* has been shown to improve thermotolerance in rice [74]. Since high temperature stress predominantly affects the membrane characteristics, membrane stability assay is commonly used to assess the stress response of plants [93,96]. Susceptibility of *NbHSP101*-silenced plants to high temperature validates the results from leaf disk experiments that showed a similar trend (Figure 6E).

## Conclusions

**Figure 7:**
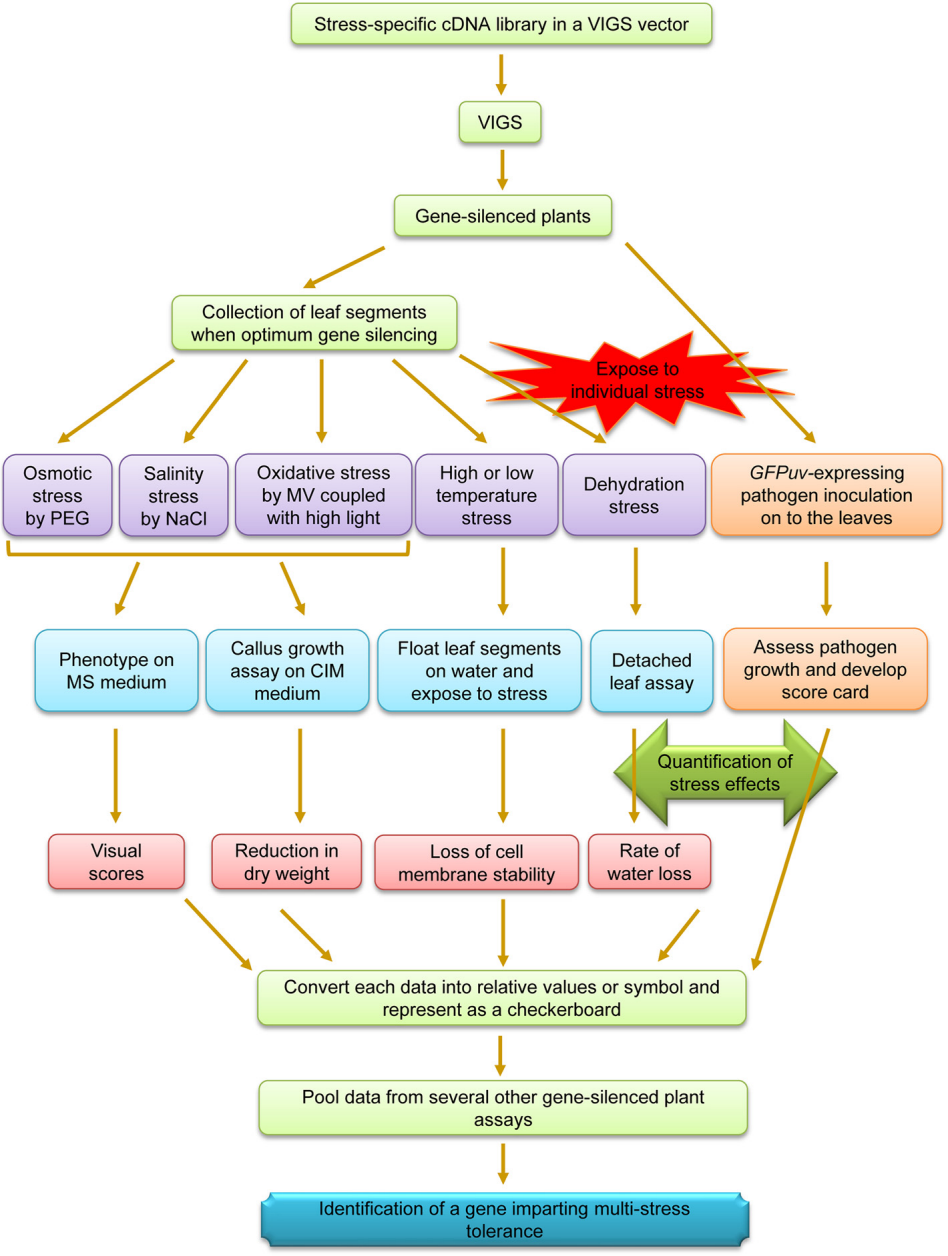
**Proposed VIGS-based high-throughput screen to identify genes involved in multi-stress tolerance in *N. benthamiana*.** A cDNA library from the plants treated with various abiotic stresses can be cloned into *pTRV2* vector. This library can be used for a forward genetic screening. TRV-VIGS can be used to silence each of these genes, and the silenced plants can be evaluated for their multi-stress tolerance by using the high-throughput methods described and demonstrated in this manuscript. Leaf disks can be collected from a silenced plant and exposed to specific abiotic stress. Such stress-treated leaf disks can be analysed for stress effects by using various physiological and biochemical assays. Similarly, the silenced plants can be inoculated with pathogens expressing *GFPuv* and their growth can be visualized by the naked eye under UV light in the dark. Based on this assay, genes involved in basal and nonhost disease resistance can be identified. Finally, relative scores can be assigned in a checkerboard for each of the stress treatments and compared with control plants. This procedure can be followed for a large number of silenced plants and thereby multi-stress tolerant plants can be identified.

During functional characterization and genetic manipulation studies of plants for stress tolerance, apart from testing a candidate gene under particular stress, it is important to screen them for broad spectrum stress tolerance [84,97]. These screens will not only help to identify the appropriate genes that impart tolerance to multiple stresses, but will also allow researchers to test susceptibility, if any, to other stresses [98]. These notions mandate quick and authoritative forward and reverse genetics assays for testing the relevance of a particular gene under multiple stresses. Despite its potential, a VIGS-based transient gene silencing system has not been used before for large-scale screening either to identify genes involved in abiotic stress tolerance or to dissect multi-stress tolerance. As depicted in Figure 7, we propose a methodology, utilizing VIGS in leaf disks combined with simple stress effect quantification methodologies, that will pave the way for such screening in the future. This methodology will facilitate identification of a single gene that can impart multi-stress tolerance or tolerance to specific stress with no negative effect on other stresses. Using this methodology, as a proof of concept, we screened 28 genes and identified genes that are important for imparting tolerance to various abiotic stresses and disease resistance. A few interesting findings from this screen which have not been shown before are the involvement of *NbRBX1* and *NbCTR1* in oxidative stress; *NtRAR1* and *NtNPR1* in salinity stress; and *NbSOS1* and *NbHSP101* in biotic stress*.* In addition to identifying genes involved in multi-stress tolerance, for the first time we demonstrate that VIGS can be effectively used to assess the relevance of genes involved in thermotolerance. The methodology described in this manuscript will be useful for large-scale testing of genes involved in thermotolerance pathways. Our results suggest the involvement of *NtEDS1*, *NbETR1*, *NbWRKY2* and *NbMYC2* in thermotolerance.

The VIGS-mediated high throughput methodology that we have described in this manuscript has several advantages to analyze the functional role of putative stress-induced genes. First, it allows precise stress imposition and quantification of stress effects using less sophisticated laboratory facilities with minimal growth chamber space and time. Second, genes can be characterized in a high-throughput manner for multi-stress tolerance using a single plant as this methodology requires less plant material. Third, experiments can be repeated multiple times since the duration of the whole experiment is shorter than whole plant assays. The limitation of this methodology is that it will only suggest the possible role of a gene under a particular stress and necessitates the study at whole plant level for further confirmation.

## Methods

### Plant growth and environmental conditions

*Nicotiana benthamiana* seeds were germinated in flats containing soilless potting mixture, Metro-Mix 830 (SUNGRO Horticulture Distribution, Inc., Bellevue, WA, USA).Three-week-old individual seedlings were transplanted into pots (10 cm diameter) containing BM7 (BFG Supply, Minneapolis, MN, USA). Fertilizer (20-10-20) solution, along with soluble trace element mix (The Scotts Co., Marysville, OH, USA), was applied as needed. Plants were grown in the greenhouse under day/night temperature of 19 ± 2°C /18 ± 2°C and photoperiod (600 μ mol m^-2^ s^-1^ light intensity) of 14 h/10 h, and relative humidity of 65%-70%.

### VIGS constructs

Total RNA was extracted from *N. benthamiana* or *N. tabacum* leaf tissues, and first-strand cDNA was synthesized using oligo-(dT)_15_ primers. Using respective gene-specific primers (Additional file 3), specific fragments (300–580 bp range) were PCR amplified and cloned into Gateway ready *pTRV2* vector [19] by following the manufacturer’s instructions (Invitrogen Corporation, Carlsbad, CA, USA). The resulting constructs were named according to the putative function of the stress gene (Table 1). TRV::*NbPDS*, TRV::*NbChlH* and TRV::*GFP* (*NbPDS*, *N. benthamiana phytoene desaturase*; *NbChlH*, *N. benthamiana Mg-chelatase H subunit*; and *GFP*, *green fluorescent protein*, whose sequence does not have any homology with the plant DNA and therefore will not cause any silencing) constructs were used as gene silencing markers and vector control [34]. Inserts within the *pTRV2* vector derivatives were confirmed by sequencing. Plasmids were mobilized into *Agrobacterium tumefaciens* strain GV2260 by electroporation. VIGS vectors were obtained from Dr. S.P. Dinesh-Kumar (University of California-Davis, USA; Additional file 4). All gene fragments used in this study were designed using siRNA scan software [99] to minimize possible off-target silencing.

### VIGS protocol

*Agrobacterium* carrying *pTRV1* or *pTRV2* derivatives were grown at 28°C in LB medium containing appropriate antibiotics. Cells were harvested from overnight grown cultures, resuspended in the inoculation buffer (10 mM MES, pH 5.5; 200 μM acetosyringone), and incubated for 2 h at room temperature in a shaker. *Agrobacterium* strains (OD_600_ = 0.5) containing *pTRV1* and one of the *pTRV2* derivatives were mixed at 1 : 1 ratio in 5 mM MES buffer (pH 5.5) and inoculated into *N. benthamiana* leaves, using a needleless syringe [19]. The inoculated plants were maintained in the greenhouse at 19 ± 2°C for effective viral infection and spread.

### Assessing the progression and persistence of gene silencing in excised leaf disks

*Progression of gene silencing*: Leaf disks (11 mm diameter) were collected from respective gene-silenced, vector control (TRV::*GFP*) and non-inoculated wild-type plants at 8 days post-inoculation (dpi) and incubated on MS medium [4.32 g of MS minimal salts (GIBCO BRL Rockville, Maryland, U.S.A), 1 ml vitamin stock solution (0.5 mg ml^-1^ nicotinic acid, 0.5 mg ml^-1^ pyridoxine, 0.5 mg ml^-1^ thiamine-HCl), 30 g sucrose, 1 g phytagel per liter of medium, pH 5.7] for a week and changes in the phenotype were recorded at two-day intervals.

*Persistence of gene silencing*: Leaf disks were collected from respective gene-silenced, vector control and non-inoculated wild-type plants at 20 dpi and incubated on MS medium or callus induction medium [CIM, MS minimal salts 4.32 g, vitamin stock 1 ml, myo-inositol 100 mg, glucose 20 g, 2, 4-D 0.5 mg, kinetin 0.3 mg, IAA 5 mg, phytagel 1 g per litre of medium, pH 5.7]. In order to prevent bacterial contamination, cefotaxime 200 μg ml^-1^ and ticarcillin 100 μg ml^-1^ were added to the medium. Persistence of the expected phenotypes was monitored for an additional 20 days. Another set of leaf disks incubated on the same CIM was harvested after 20 days culturing and used for RNA isolation to analyze respective endogenous transcript levels of silenced genes. Semi-quantitative RT-PCR was performed using specific primers (Additional file 3).

### Stress treatment for excised leaf disks

Leaf disks (11 mm diameter) were excised from respective gene-silenced, vector control and non-inoculated wild-type plants grown under non-stress conditions at 20 dpi and exposed to various abiotic stresses as described below.

*Osmotic stress*: Leaf disks were incubated on MS medium supplemented with 10% PEG-10000 (Sigma Aldrich Inc., St. Louis, MO, USA), and the phenotypic changes were observed at 15 days after incubation. To assess PEG-induced osmotic stress effects on callus growth in silenced plants, leaf disks were incubated on CIM supplemented with 10% PEG-10000. Increase in growth of leaf disks was assessed by measuring dry weight at 20 days after incubation. PEG plates were prepared according to a previously published protocol [60]. Osmotic stress protocols that we used were slightly modified from protocols described previously [100,101].

*Salinity stress*: Leaf disks were incubated on MS medium supplemented with 200 mM NaCl, and the phenotypic changes were observed at 15 days after incubation. To measure the effect of salt stress on callus growth, leaf disks were incubated on CIM supplemented with either 100 mM or 200 mM NaCl. Increase in growth of leaf disks was measured at 20 days after incubation. Stress protocol that we used for callus growth assay was similar to the previously established protocol [100,102].

*High temperature stress*: Leaf disks were floated on deionized water and exposed to an acclimation temperature of 35°C for 6 h and then to the severe temperature of 45°C for 1 h. Cell membrane stability (CMS) was assessed at the end of the stress period as described previously [60].

*Low temperature stress*: Leaf disks were floated on deionized water and exposed to an acclimation temperature of 4°C for 12 h and then to the severe low temperature of -2°C for 1 h. CMS was assessed at the end of the stress as described previously [60].

*Oxidative stress*: Leaf disks were incubated on MS medium supplemented with 10 μM menadione sodium bisulfite (Sigma Aldrich Inc., St. Louis, MO, USA), and phenotypic changes were observed at 15 days after incubation. To study the influence of oxidative stress on callus growth, leaf disks were incubated on CIM supplemented with 10 μM menadione sodium bisulfite for 20 days. Reduction in callus dry weight was measured at the end of the stress period.

### Quantification of rate of water loss by detached leaf assay

This assay was performed for assessing the dehydration avoidance of gene-silenced plants by measuring the rate of water loss in detached leaves. Leaves were detached from respective gene-silenced, vector control and non-inoculated wild-type plants, and air dried on the bench under controlled environmental conditions (temperature 20°C, light 100 μmol m^-2^ s^-1^ and relative humidity 30-35%). Reduction in fresh weight over time was measured at 1 h intervals over a 6 h period, and water loss rate was calculated using the formula given below. This assay was modified from a previously published protocol [60].

Rate of water loss (%) = (Fresh weight at time ‘zero’/Fresh weight at time ‘n’) x 100

### Whole plant stress treatment

A new batch of respective gene-silenced, vector control and non-inoculated wild-type plants were grown in the greenhouse under non-stress conditions and subjected to the following stresses.

*Water deficit stress*: Plants were exposed to gradual water deficit stress by maintaining them at 80% field capacity (FC) for 5 days, followed by 60% FC for 5 days and 50% FC for 5 days [103]. The amount of water lost through evapotranspiration was replenished by weighing the pots daily at a fixed time of the day. Stress responses were quantified at the end of the stress period by assessing reduction in photosynthetic rate. This method is a modification from a previously published protocol [32].

*Salinity stress*: Plants were exposed to gradual salt stress by irrigating them with 100 mM NaCl solution for 5 days followed by 200 mM solution for 5 days and then 300 mM solution for 5 days. Salinity stress responses were quantified at the end of the stress period by measuring the reduction in photosynthetic rates.

*High temperature stress*: To assess thermotolerance, plants were exposed to high temperature stress under controlled environmental conditions in a growth chamber. Plants were initially acclimated by exposing them to 35°C for 6 h and then to the challenging temperature of 45°C for 1 h; stress responses were quantified by measuring CMS [96].

### Pathogen assays

*Pseudomonas syringae* strains were grown in King’s B medium; *Xanthomonas campestris* pv. *vesicatoria* was grown in LB medium at 30°C. Bacterial cells were collected by centrifugation of overnight grown culture at 2,700 g for 10 min, washed twice in sterile water, and resuspended in sterile water to the desired concentrations. The bacterial suspensions were spot-inoculated at a concentration of 1 × 10^5^ colony forming units (CFU) onto the fully expanded third leaf from the top of the respective gene-silenced *N. benthamiana* plants, three weeks after TRV inoculation, using a needleless syringe as described previously [87]. Growth of host pathogen *P. syringae* pv. *tabaci* and nonhost pathogens *P. syringae* pv. *tomato* T1 and *X. campestris* pv. *vesicatoria* was observed in gene-silenced plant leaves using respective *GFPuv*-expressing bacteria [85], in addition to visual observations on disease symptom development.

### Semi-quantitative RT-PCR

*Confirmation for presence of virus*: RT-PCR was performed to determine the presence of TRV in the inoculated plants. Total RNA was isolated from newly developed upper non-inoculated leaves, and virus-specific cDNA was synthesized using *TRV-*coat protein specific reverse primer (Additional file 3).

*Confirmation of endogenous gene transcript levels by RT-PCR*: To quantify the respective endogenous transcript levels in silenced plants, RT-PCR was performed using specific primers given in the Additional file 3. Total RNA was isolated from newly developed upper non-inoculated leaves, and RNA samples were treated with RNAse-free DNAse prior to reverse transcription reaction using the RT-PCR Kit (TURBO™ DNase, Invitrogen, Grand island, NY, USA). First-strand cDNA was synthesized by reverse transcribing 2 μg of total RNA (Omniscript RT kit; Qiagen Inc, Valencia, CA, USA) using oligo-dT_(15)_ primers, and RT-PCR was performed. To study the down-regulation of transcripts, PCR primers that anneal outside the region targeted for silencing were used to ensure that only endogenous genes would be tested. The *elongation factor*-*1 alpha* of *N. benthamiana* (*NbEF1*α) was used as an internal control.

### Estimation of cell membrane stability

Leaf disks (11 mm in diameter) were rinsed in deionized water to remove the solutes leaked at the cut ends and then incubated in deionized water for 8 h at 25°C under constant shaking (25 rpm). The conductivity of electrolytes that leaked into water from stressed leaf samples was recorded (T1) using conductivity bridge (Orion pHuture MMS555A; Thermo Electron Corporation, Beverly, MA, USA). Subsequently, leaf disks were boiled for 30 min, and a final reading was recorded (T2) after cooling to room temperature. Similarly, conductivity was also measured using non-stressed samples (C1 and C2). The membrane stability was calculated using the below formula [73].

$$\text{Cell}\;\text{membrane}\;\text{stability}\;\left(\text{CMS}\right)=\frac{\left[1,-,\left(\frac{T1}{T2}\right)\right]}{\left[1,-,\left(\frac{C1}{C2}\right)\right]}\times 100$$

### Measurement of photosynthetic rate

Photosynthetic rates (μmol m^-2^ s^-1^) were measured on the third leaf down from the top using a portable photosynthesis system (LI-6400; LI-COR, Lincoln, NE, USA) at an ambient CO_2_ concentration (370 μmol mol^-1^) and 1,000 μmol m^-2^ s^-1^ light intensity using LICOR light source at a chamber temperature of 28°C. The values were expressed as percent reduction over non-stressed plants.

### Statistical analysis

The data points were analyzed for significant differences in stress effects on each gene-silenced plants when compared to wild-type and vector control (TRV::*GFP*) plants (*P* < 0.05) by Student’s *t*-test. One-way ANOVA (generalized linear model procedure; SAS software, SAS Institute Inc., Cary, NC, USA) analysis was performed to test the significant difference in water loss between time points within each gene-silenced plant. The statistical significance of values in graphs was indicated either as asterisks (*t*-test) or as letters (ANOVA). Unless otherwise specified, values not significantly different from wild-type and vector control plants were not shown in figures.

## Abbreviations

ADR1: *Activated disease resistance 1*; APX3: *Ascorbate peroxidase 3*; BIP5: *Binding protein 5*; CAT3: *Catalase isozyme 3*; CBL1: *Calcineurin B-like protein 1*; ChlH: *Mg-chelatase H subunit*; CIM: Callus induction medium; CMS: Cell membrane stability; CTR1: *Constitutive triple response 1-like protein kinase*; CYCD2: *Cyclin D2.1 protein*; DHAR1: *Dehydroascorbate reductase 1*; dpi: Days post-inoculation; EDS1: *Enhanced disease susceptibility 1*; EF1α: *Elongation factor-1 alpha*; EST: Expressed sequence tag; ETR1: *Ethylene response 1*; FC: Field capacity; FER2: *Ferritin 2*; FLS1: *Flavonol synthase 1*; GBP16: *Putative monomeric G-protein 16*; GFP: Green fluorescent protein; GPX2: *Glutathione peroxidase 2*; GST1: *Glutathione S-transferase 1*; HSP101: *101 kDa Heat shock protein*; MC: *Metacaspase type II*; MEK1: *MAP kinase/ERK kinase 1*; MYB1: *MYB-related transcription factor 1*; MYC2: *MYC-related transcription factor 2*; Nb: *Nicotiana benthamiana*; NPR1: *Non-expresser of pathogenesis related gene 1*; Nt: *Nicotiana tabacum*; P5CS1: *Delta-1-pyrroline-5-carboxylate synthetase 1*; PAL1: *Phenylalanine ammonia-lyase 1*; PDS: *Phytoene desaturase*; PEG: Polyethylene glycol; Pstab: *Pseudomonas syringae* pv*. tabaci*; PstT1: *Pseudomonas syringae* pv. *tomato* T1; PTGS: Post-transcriptional gene silencing; RAR1: *Required for Mla12 resistance 1*; RBX1: *Ring box 1 like protein*; SOS1: *Salt overly sensitive 1*; TRV: *Tobacco rattle virus*; VIGS: Virus-induced gene silencing; WRKY1: *WRKY DNA-binding protein 1*; WRKY2: *WRKY DNA-binding protein 2*; XCV: *Xanthomonas campestris* pv. *vesicatoria*.

## Competing interests

The authors declare that they have no competing interests.

## Authors’ contributions

RV performed all abiotic stress experiments, analyzed the data, prepared figures and drafted background and methods sections. MS and KSM designed the study and coordinated the experiments. MS performed pathogen assay/data analysis. RV, MS and KSM wrote the manuscript. MU edited the draft manuscript. All authors read and approved the final manuscript.

## Supplementary Material

Additional file 1 **RT-PCR showing reduction in endogenous transcript levels of four selected stress-responsive genes in silenced plants.** Total RNA was extracted from wild-type-, TRV::*GFP-* (vector control), TRV::*NbSOS1-*, TRV::*NbP5CS1-*, TRV::*NbHSP101-* and TRV::*NtEDS1*-inoculated plant leaves at 0 and 20 dpi. A) Semi-quantitative RT-PCR was performed using first-strand cDNA as a template with 30 PCR cycles using respective gene-specific primers, and the PCR product was resolved on agarose gel. dpi, days post-infiltration; GOI, gene of interest; *EF1α*, *Elongation factor 1 alpha* as loading control. B) The band intensity of RT-PCR products was analyzed using ImageJ software version 1.34 s (National Institutes of Health) to calculate the reduction in transcript levels in silenced plants keeping the expression levels of wild-type plants as one. Click here for file

Additional file 2 **Representative phenotypes used in abiotic stress and pathogen susceptibility scoring.** A) Phenotype of TRV::*NbSOS1*-silenced leaf disks. Leaf disks were incubated on MS medium supplemented with 200 mM NaCl, and the photograph was taken 15 days after incubation on stress medium. B) Response of gene-silenced plants to *Pseudomonas syringae* pv. *tomato* T1 inoculation. TRV::*NbGPX*, vector control and wild-type *N. benthamiana* leaves were inoculated with a nonhost pathogen, *P. syringae* pv. *tomato* T1, at a concentration of approximately 1 x 10^5^ cfu ml^-1^ by using a needleless syringe. Leaves were photographed at 5 dpi. Click here for file

Additional file 3 Primers used in the study. Click here for file

Additional file 4 **TRV-based VIGS constructs.***Tobacco rattle virus (*TRV*)* cDNA clones are placed in between the duplicated CaMV35S promoter (2X35S) and nopaline synthase terminator (NOSt) in a T-DNA vector. LB and RB, left and right borders of T-DNA; RdRp, RNA-dependent RNA polymerase; MP, movement protein; 16 K, 16 kDa cysteine rich protein; CP, coat protein; and Rz, self-cleaving ribozyme. A) TRV RNA1-based viral vector, plasmid of 6.791 kb [NCBI# AF406990], is referred to as *pTRV1*. B) TRV RNA2-based viral vector, plasmid of 9.663 kb [NCBI# AF406991], is referred to as *pTRV2*. It’s a modified vector compatible for Gateway recombination. The gene fragments were cloned into *pTRV2* by replacing the *ccdB* gene, and these derived constructs were used for silencing studies. Details of this vector were described previously [31]. Click here for file
