# Supplementary figures and images for: A high-throughput virus-induced gene silencing protocol identifies genes involved in multi-stress tolerance
Source: BMC Plant Biol. 2013 Dec 1;13:193. doi: 10.1186/1471-2229-13-193 (PMC3879149; doi:10.1186/1471-2229-13-193)

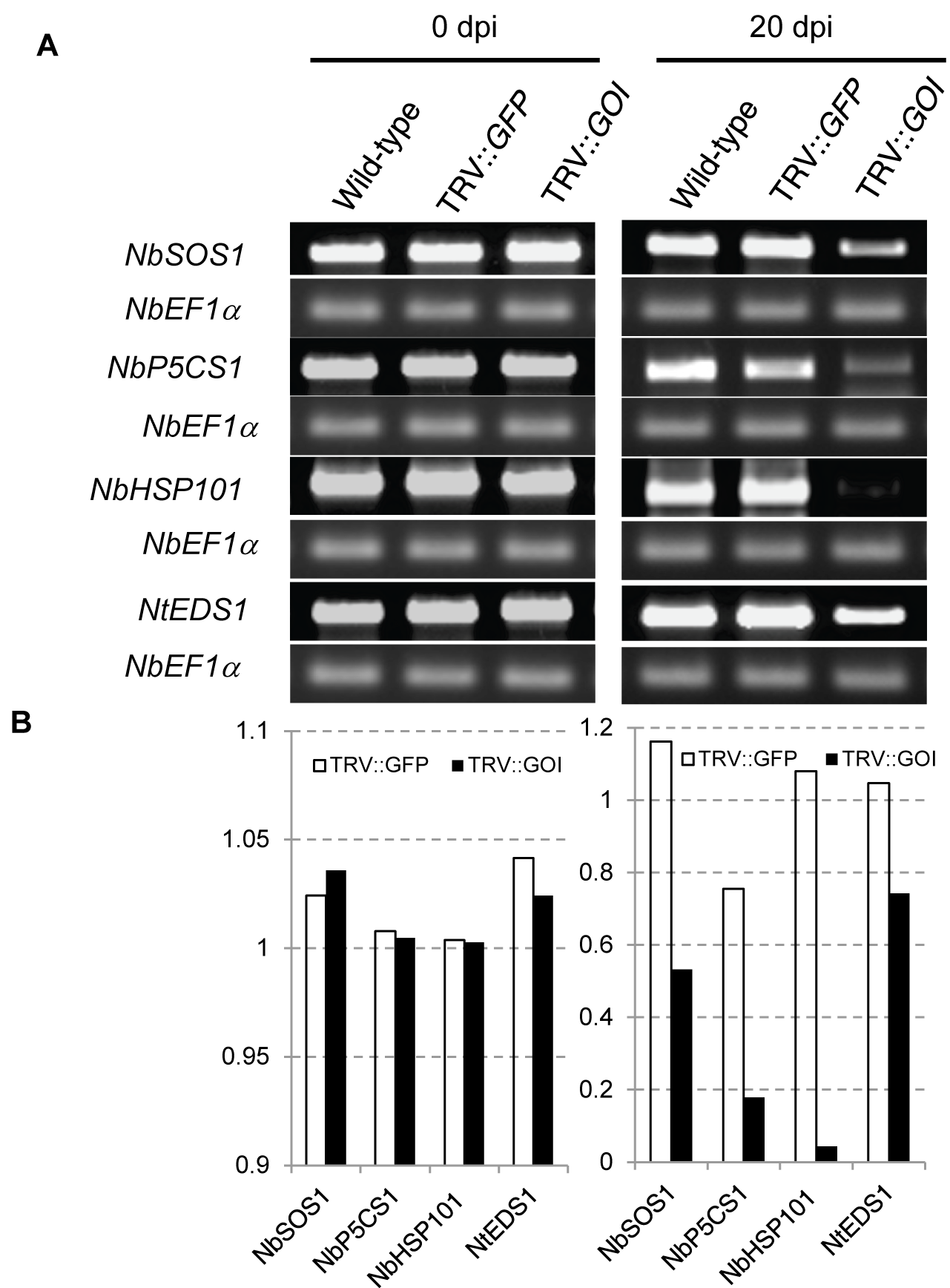

Supplement: Additional file 1 — RT-PCR showing reduction in endogenous transcript levels of four selected stress-responsive genes in silenced plants. Total RNA was extracted from wild-type-, TRV::GFP- (vector control), TRV::NbSOS1-, TRV::NbP5CS1-, TRV::NbHSP101- and TRV::NtEDS1-inoculated plant leaves at 0 and 20 dpi. A) Semi-quantitative RT-PCR was performed using first-strand cDNA as a template with 30 PCR cycles using respective gene-specific primers, and the PCR product was resolved on agarose gel. dpi, days post-infiltration; GOI, gene of interest; EF1α, Elongation factor 1 alpha as loading control. B) The band intensity of RT-PCR products was analyzed using ImageJ software version 1.34 s (National Institutes of Health) to calculate the reduction in transcript levels in silenced plants keeping the expression levels of wild-type plants as one. [file 1471-2229-13-193-S1.tiff]

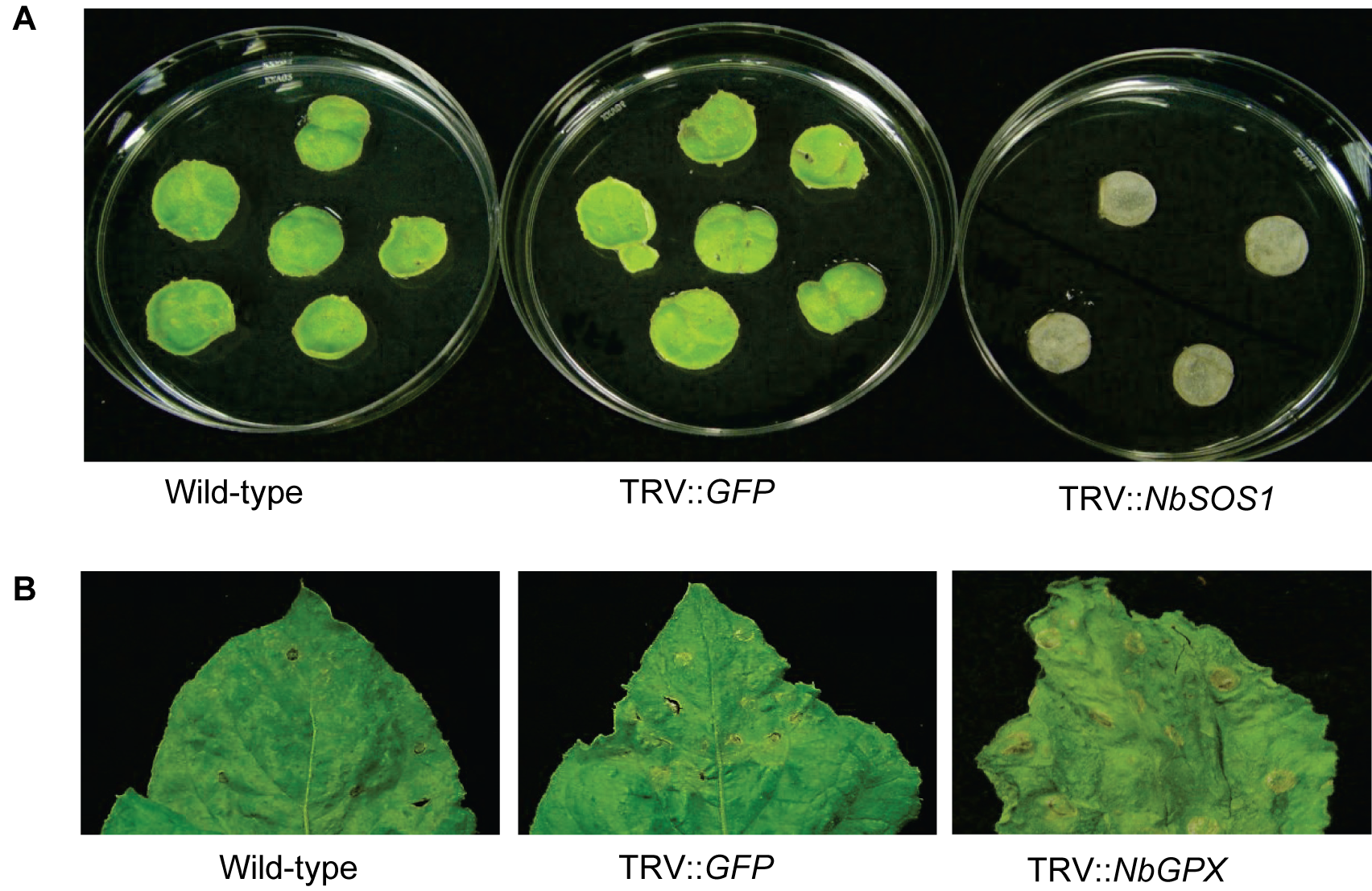

Supplement: Additional file 2 — Representative phenotypes used in abiotic stress and pathogen susceptibility scoring. A) Phenotype of TRV::NbSOS1-silenced leaf disks. Leaf disks were incubated on MS medium supplemented with 200 mM NaCl, and the photograph was taken 15 days after incubation on stress medium. B) Response of gene-silenced plants to Pseudomonas syringae pv. tomato T1 inoculation. TRV::NbGPX, vector control and wild-type N. benthamiana leaves were inoculated with a nonhost pathogen, P. syringae pv. tomato T1, at a concentration of approximately 1 x 10^5^ cfu ml-1 by using a needleless syringe. Leaves were photographed at 5 dpi. [file 1471-2229-13-193-S2.tiff]

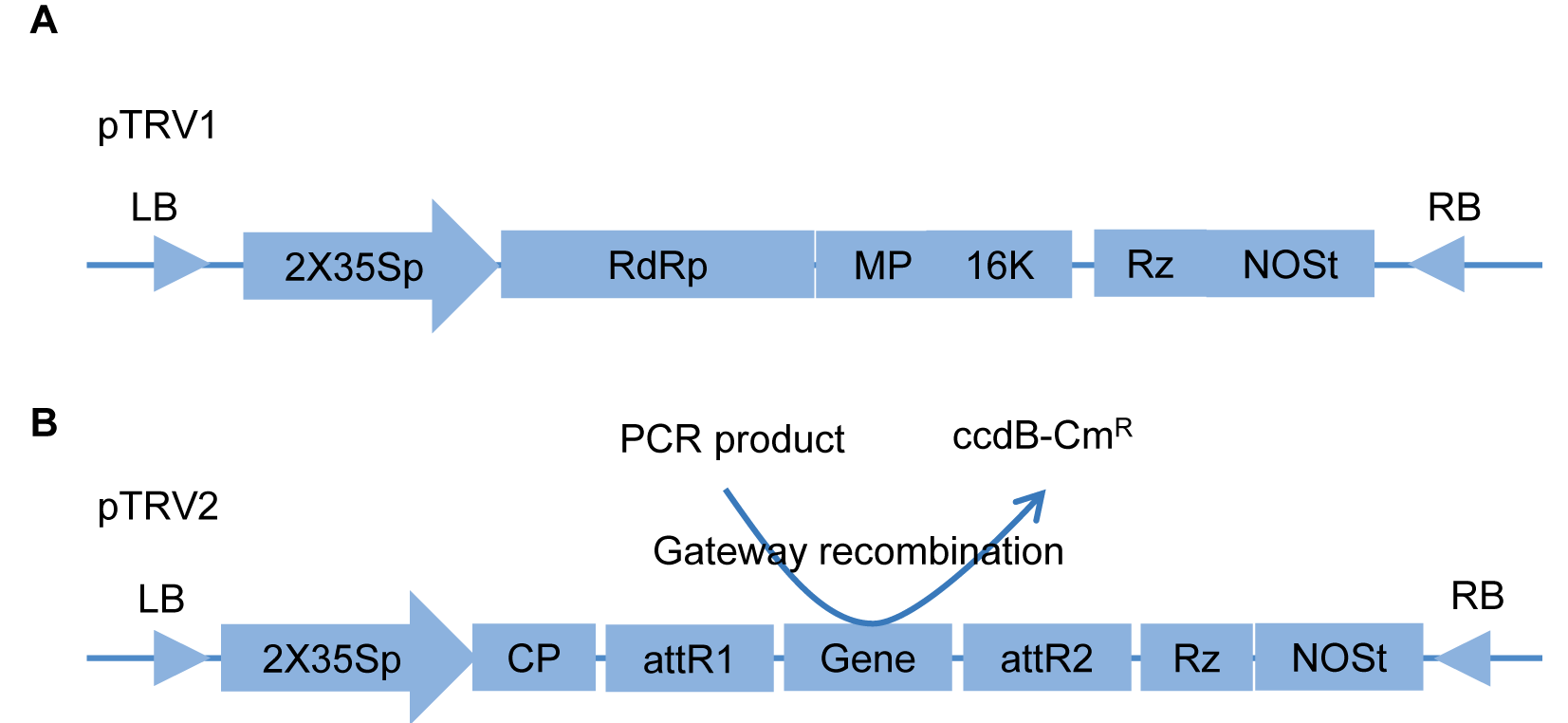

Supplement: Additional file 4 — TRV-based VIGS constructs.Tobacco rattle virus (TRV) cDNA clones are placed in between the duplicated CaMV35S promoter (2X35S) and nopaline synthase terminator (NOSt) in a T-DNA vector. LB and RB, left and right borders of T-DNA; RdRp, RNA-dependent RNA polymerase; MP, movement protein; 16 K, 16 kDa cysteine rich protein; CP, coat protein; and Rz, self-cleaving ribozyme. A) TRV RNA1-based viral vector, plasmid of 6.791 kb [NCBI# AF406990], is referred to as pTRV1. B) TRV RNA2-based viral vector, plasmid of 9.663 kb [NCBI# AF406991], is referred to as pTRV2. It’s a modified vector compatible for Gateway recombination. The gene fragments were cloned into pTRV2 by replacing the ccdB gene, and these derived constructs were used for silencing studies. Details of this vector were described previously [31]. [file 1471-2229-13-193-S4.tiff]
