# Supplementary material for: A high-throughput virus-induced gene silencing protocol identifies genes involved in multi-stress tolerance
Source: BMC Plant Biol. 2013 Dec 1;13:193. doi: 10.1186/1471-2229-13-193 (PMC3879149; doi:10.1186/1471-2229-13-193)
Supplement: Additional file 3 — Primers used in the study. [file 1471-2229-13-193-S3.pdf]

	Gene name	Primer sequence (5′-3′)	Product size
**A. Primers used for amplification of *Nicotiana benthamiana* or *Nicotiana tabacum* homologs for cloning into *pTRV2* vector following Gateway technology**			
1	*NbSOS1*	-TCTTTCAGCTCCTGCACCGTTACT-<br>-TGACCAGTTCGTTCCACATCCTGT-	302
2	*NbAPX*	-ACGAAAGATGGTCCAGGAAACCCT-<br>-TCGGAAAGCTCTCTCTTTCCGGTT-	323
3	*NbGST*	-GCATGCAAAGGGAGAGTGTTGCTA-<br>-ACTCCTCAAGTTGGCATCCTCCAA-	321
4	*NbCAT3*	-TGGAGTCAAGTGCTTGTTGGAGGA-<br>-AAACACCAGGGACAACAATGGCAG-	281
5	*NbDHAR*	-AGGGCACTTCTGACATTGGAGGAA-<br>-ACATATGGTCCGTGAGCCTTGAGA-	318
6	*NbGPX*	-AATGTTGCATCACAGTGTGGCCTG-<br>-TGGTTGGAGAGTAGCGATCAACGA-	321
7	*NbFER2*	-GCATGCTGTAGCCACACGAAACAA-<br>-AGCACTGGGACTTTCCATCACCAA-	312
8	*NbHSP101*	-TGATCCTCTGTCCCACAAGCAGTT-<br>-TCACAAGCCCTCCATTCTTCTCCA-	277
9	*NbBIP5*	-TTTGATGGCAAGGAGCCCAACAAG-<br>-AGCAGTCTGCAGTCCTTTGTGAGA-	282
10	*NbP5CS1*	-TGTTGAAAGGAGGAAAGGAGGCCA-<br>-CATTGCATTGCAGGCTGCTGGATA-	305
11	*NbCBL*1	-ATGGGCTGTTTCCAGTCTACAGCA-<br>-TCTTCTTGTGAGGCATTTGGGTGG-	392
12	*NbMYC2*	-ATTCTTTTTCATATACTTTATATAG-<br>-GGGAAGGAAGGGAAATCGCCG-	378
13	*NbGBP16*	-CGATGAGACGTCACTGGAAGAAG-<br>-CGAATTGAGACGGGAAAAATAAG-	426
14	*NbRBX1*	-GACTGTTAGACGGAATATTCCC-<br>-TTGTCAGTCAAAATAGCACCTTATC-	382
15	*NbPAL*1	-TCTTCGAACATCTCCACAGTGGCT-<br>-TGCAATTTCAGCACCCTTGAGTCC-	305
16	*NtEDS1*	-GAGTATCAGACCAAGTGTGATATCCG-<br>-GCTGAGGTGGGAGTGTTTTCCACC-	548
17	*NtNPR1*	-GAAAGAGCCTAAAATTGTAGTGTC-<br>CTATTTCCTAAAAGGGACCTTATT-	753
18	*NtRAR1*	-GTGCCATCCTTTGGTGCATGGAGG-<br>-AGGAAAGCACACAACAGAAAAACC-	468
19	*NbADR1*	-AGGGCTTCCTTTGGCTCTTAAGGT-<br>-TCTCCAGCTCGTGCATCTTTGACT-	320
20	*NbMC*	-ATCCTTGCCTCTTTCCACCCTCAT-<br>-CCGGCATAAACCTCTTGTTTGCCT-	285
21	*NbCYCD2*	-GTGCAATTGTTAGCTGTGGCCTGT-<br>-CAGACATTGCCACTGATGCTGCAA-	310
22	*NbCTR1*	-CGGGAATTCGTTGCAATTATGAAGCGGTTGCG-<br>-CGGCTCGAGTCATGAGAGCAACTGCATGTCTGT-	369
23	*NbWRKY1*	-CGGGAATTCGGATGATACTCCACCTATTAAGTC-<br>-CGGCTCGAGTGCACCAATATGAACAACCATGTC-	538
24	*NtMEK1*	-AGATCTTCAGTTCTCATTGGAAGATCTTGA-	347

		-CGGTCTAGAGTAAAACCTGCTTGCAAACAACTGC-	
25	*NbWRKY2*	-CGGGAATTCGACCAAGTCTCCGGAACAAGTGA-<br>-CGGCTCGAGCGTAGCAGTGCTATAGGTTGCTG-	512
26	*NbETR1*	-AATACATGCCTGGTGAGGTGGT-<br>-ACGTTGCTCCGGAGTTAGATCA-	-
27	*NbFLS*	-GCTCATCACTTGGGCTTGGGTTAGAAG-<br>-TGGATTGGCCTCATTAACCAGCTTAGG-	416
28	*NbMYB1*	CGGGAATTCATGGTGAGAGCTCCTTGTTGTGAGAAAATG-<br>CGGCTCGAGGCACTGGGCTATCAATGTGTTTTTGTGT-	298
**B. Primers used in RT-PCR analysis**			
29	*NbSOS1*	-GTTCAAGCAGCATACTGGGAAATG-<br>-CAAGCTTCCGGTGGACAAAAC-	199
30	*NbP5CS1*	-GATGTGATTGATCTTGTTATACC-<br>-CCTTATGAACGAGAAGAGTTTC-	217
31	*NbHSP101*	-GGAAGGCCACAGCAACCAAC-<br>-CAGAGTGTTGTTCCATGTAC-	154
32	*NbGPX*	-ATGACGGTTCAACATTGTCAC-<br>-CTTACACTTCACACTTACATAG-	181
33	*NtEDS1*	-GAGAAGTGAAAGATGAGCTG-<br>-CTACTGTACCAACCATCAAC-	160
34	*NbPDS* (NCBI accession # DQ469932)	-GCATTTTGATTGATTGCTTTGAAC-<br>-CACAATCGGCATGCAAAGTC-	101
35	*NbChlH* (NCBI accession # AF014051)	-TGCAGTGGAGAAAGAAAGGGAC-<br>-CCCCACAACCTCAAAAGAAGC-	190
36	*TRV2-CP*	-CTGGGTTACTAGCGGCACTGAATA-<br>-TCCACCAAACTTAATCCCGAATAC-	-
37	*NbEF1α*	-CACGCATTGCTTGCTTTCA-<br>-TCCATCTTGTTACAGCAGCAAATC-	220
